# Demographics of Rhesus Phenotype of Blood Donors in Calabar: A Case Study of University of Calabar Teaching Hospital, Calabar, Cross River State, Nigeria

**DOI:** 10.1155/2020/2659398

**Published:** 2020-08-27

**Authors:** Joyce Ezekiel Etura, Rose A. Amaechi, Josephine O. Akpotuzor, Henshaw Uchechi Okoroiwu

**Affiliations:** ^1^Haematology Unit, Department of Medical Laboratory Science, University of Calabar, Calabar, Nigeria; ^2^Haematology Unit, Department of Medical Laboratory Science, Ambrose Alli University, Ekpoma, Edo State, Nigeria

## Abstract

**Background:**

Rhesus antigens have been documented to cause haemolytic disease of the newborn as well as acute and delayed transfusion reactions. This study was performed to evaluate the frequency of rhesus antigens (C, c, D, E, and e) in the studied population.

**Method:**

This study was a cross-sectional study involving 130 prospective blood donors attending University of Calabar Teaching Hospital (UCTH) donor clinic. Donors were grouped for Rh antisera (anti-E, anti-e, anti-C, anti-c, and anti-D) using the standard serologic technique.

**Result:**

The most prevalent Rh antigen was “c” (98.5%), followed by “D” (97.7%), while the least was “C” (30.7%). The most prevalent phenotype was cDe/cDe (R_0_R_0_).

**Conclusion:**

This work therefore concludes that the most prevalent rhesus antigen and rhesus phenotype was c and cDe/cDe among blood donors in University of Calabar Teaching Hospital.

## 1. Background

The term “blood group” refers to the entire blood group system comprising red blood cell antigens whose specialty is controlled by a series of genes which can be allelic or linked very closely on the same chromosome [1]. Presently, 33 blood group systems representing over 300 antigens are listed by International Society of Blood Transfusion [2, 3]. The antigens may occur as integral proteins, where the polymorphism lies in the variation in amino acid sequence (e.g., rhesus (Rh) and Kell) or as glycoproteins or glycolipids (e.g., ABO) [1]. The phenotype of blood group of an individual is the observable expression of the genes inherited by the person and reflects the biologic activity of genes. The presence or absence of antigens on red cells is determined by serological testing representing the phenotype [4, 5].

The rhesus blood group, formerly known as the rhesus system, is the second most important blood group system after ABO [6]. At a more comprehensive level, the rhesus system is considered as a gene complex that gives rise to various combinations of three alternative antigens C or c, D or d, and E or e, as originally suggested by Fisher [7]. The rhesus locus is located on chromosome 1 and comprised two highly homologous, very closely linked genes RhD, C, and E [8]. This concept of D, C, c, E, and e genes linked closely and transmitted together is consistent with Fisher nomenclature, and it is recommended by the World Health Organization Expert Committee in the interest of simplicity and uniformity [9]. The rhesus antigens are defined by corresponding antisera with the exception of “anti-d,” which does not exist as it was thought to be amorphic without any corresponding antigen on red blood cells [8]. Anti-D is the most immunologically and clinically most important antibody in the rhesus system causing haemolytic transfusion and haemolytic disease in the newborn [10–12]. However, antibodies to the other Rh phenotype C, c, E, and e, to a lesser extent, cause haemolytic disease in the newborn and transfusion haemolytic reaction [10, 13, 14]. In routine transfusion practice in our study environment, Rh antigen typing is restricted to only D phenotype screening partly due to unavailability of antisera: C, c, E, and e and lack of policy to this effect [15]. Women of child bearing age and patients prone to recurrent blood transfusion in our environment and similar settings are at higher risk of developing haemolytic disease in the newborn and haemolytic transfusion reaction due to antibodies against these nonscreened Rh antigens, at times fatal [13, 14]. Most of the studies done in the country (Nigeria) are restricted to the D phenotype. This study is aimed at filling this gap by providing the information on the prevalence of Rh phenotypes among blood donors as a baseline for policy formation and future planning towards safe blood transfusion and prevention of haemolytic disease of the newborn.

## 2. Methods

This study took a cross-sectional design with a systematic random sampling technique. A total of 130 prospective blood donors attending University of Calabar Teaching Hospital (UCTH) donor clinic were recruited into the study. Two millimeters (2 ml) of venous blood was collected from each of the 130 blood donors through veinpuncture using the antecubital vein into a plain container. The rhesus phenotypes were determined according to manufacturer's instructions using five specific monoclonal antisera (anti-D, anti-E, anti-C, anti-c, and anti-e) supplied by Lorne Laboratories (United Kingdom). The principle is based on the ability of Lorne reagents to cause a direct agglutination of the test red blood cells that carry the corresponding rhesus antigen. The presence of the group-specific rhesus antigen was indicated by agglutination.

## 3. Result

Of the five major antigens screened, “c” antigen was found to be the most common antigen (98 : 5%; *n* = 128), followed by the “D” antigen (97.7%; *n* = 127) and “e” antigen (95.4%; *n* = 124). The least observed antigen was “C” (30.7%; *n* = 40), whereas antigen “E” had a prevalence of 39.2% (*n* = 51) (Table 1).

The cDe/cDe phenotype had the highest distribution (46.2%; *n* = 60), followed by cDE/cde (20.6%; *n* = 26). The least observed phenotypes were Cde/cde, cde/cde, and CDE/CDe with a prevalence of 0.8% (*n* = 1) (Table 2).

Table 3 shows the frequency of rhesus antigen observed in this study compared with published results in other parts of Nigeria and sub-Saharan Africa.

Table 4 shows the comparison of the rhesus phenotype distribution in this study with other published data from studies in Nigeria.

## 4. Discussion

Data on various blood group antigens and phenotype frequencies in a population are essential in work-up plan for blood transfusion services [5].

We observed rhesus antigen “c” to be the most prevalent antigen with a prevalence of 98.5%. This finding is similar to the report of Jeremiah and Buseri and Jeremiah and Odumody [16, 17] who reported the same antigen as most prevalent (99.8% and 100%, respectively) in studies done in Port Harcourt and Calabar, Nigeria. Antigens D (97.7%) and e (95.4) were the next common frequently occurring antigens, respectively. In contrast with the present study, both studies recorded a higher prevalence of antigen “e” than “D” [16, 17]. However, the observation of this study is at variance with the report of Gwaram and Abdullah [15] who reported highest occurrence of antigen “D” in a study in Kano, Nigeria. Studies in other African countries such as Mauritania [18] reported antigen “e” (98.2) as the most prevalent rhesus antigen, while the study in Cote d'Ivoire [19] reported both antigens “c” (99.9%) and “e” (99.9%) as the most prevalent rhesus antigens. More so, studies outside Africa has reported antigen “e” (98.4%) in India [5], antigen “D” 99.0% in China, and antigen “e” (98%) in Black Americans as the most frequently occurring antigens. The disparity in the studies in Nigeria may be due to ethnic variations owing to heterogeneous nature of Nigeria. Nigeria has been described as a heterogeneous society with ethnic pluralism [20]. Rhesus antigens have been documented to vary among races [21]. Anti-D, anti-C, anti-E, and anti-c have all been implicated in haemolytic transfusion reactions, particularly delayed reactions [22]. Anti-D causes the most severe form of haemolytic disease of the newborn, and it is the major cause of fetal death. Another alloantibody capable of causing severe HDN includes anti-c [23, 24].

In our study, the most common phenotype was cDe/cDe (Dccee; R_0_R_0_) followed by cDE/cde (DccEe; R_2_r). This finding is similar to previous studies in Port Harcourt and Calabar [16, 17] that reported Dccee (cDe/cDe) as the most prevalent phenotype (60.8% and 73.61%, respectively). Other studies outside Nigeria have reported CDe/CDe (R_1_R_1_), CDe/cDe (R_1_R_0_), and cDe/cDe (R_0_R_1_) as the most prevalent phenotypes in Asians [25, 26], Caucasians [27], and Black Americans [25], respectively. However, cDE/cDE (R_2_R_2_), CdE/cDe (r^y^R_0_) cdE/cde (r^11^r), cdE/cdE (r^11^r^11^), Cde/Cde (r^1^r^1^), CdE/Cde (r^y^r^1^), CdE/cdE (r^y^r^11^), CdE/cde (r^y^r), and CdE/CdE (r^y^r^y^) were not detected in the studied population. Due to the low sample size, we could not declare these phenotypes as rare blood. Rare blood is defined on the basis of blood group characteristics, as being found at a frequency of <1 : 1000 random samples in a given population [28, 29].

The prevalence of RhD positive cases was 97.7% and *n* = 127, whereas the RhD negative was 2.3% (*n* = 3). This finding is similar to 97.1% and 93.9% reported in Kano [15] and Benin in Nigeria, respectively [30].

## 5. Conclusion

The prevalence of rhesus antigens and phenotypes showed similar pattern in this study and other previous studies with antigen c and cDe/cDe phenotypes occurring as most prevalent antigen and phenotype in Nigerians and other African population. The antigens were found in the order c > D > e > E > C. The data of these antigens put the call to incorporate their testing in synergy with the D antigen in routine blood screening prior to transfusion. We observed low frequency of rhesus D negative blood group in this study (2.3%). The low prevalence of RhD negative blood groups in our study environment also illuminates the need to make proactive plan in event of RhD negative patient requiring blood transfusion. Our frequency data on Rh antigens can help to implement different transfusions and obstetric strategies, which can ultimately improve our patient care. Mass scale typing, however, might be required to complete the database for Rh antigens in Nigeria.

## Figures and Tables

**Table 1:** Distribution of major rhesus antigens among the studied population.

Rh antigen	ISBT nomenclature	Number positive (%)	Number negative (%)
C	RH2	40 (30.7)	90 (69.2)
c	RH4	128 (98.5)	(1.5)
D	RH1	127 (97.7)	3 (2.3)
E	RH3	51 (39.2)	79 (60.7)
e	RH5	124 (95.4)	6 (4.6)

Rh = rhesus; ISBT = International Society of Blood Transfusion.

**Table 2:** Distribution of rhesus phenotypes in the studied population.

Rhesus phenotype		Frequency (%)
Fisher notation	Weiner shorthand	
Rhesus positive		
cDe/cDe	R_0_R_0_	60 (46.2)
cDe/CDe	R_0_R_1_	16 (12.3)
cDE/cde	R_2_r	26 (20.6)
CDe/CDe	R_1_R_1_	1 (0.8)
cDE/Cde	R_2_r^1^	18 (13.8)
cDE/cDE	R_2_R_2_	—
cDE/CDE	R_2_R_z_	3 (2.3)
CDE/CDE	R_z_R_z_	1 (0.8)

Rhesus negative		
Cde/cde	r^1^r	1 (0.8)
cde/cde	Rr	1 (0.8)

**Table 3:** Rhesus antigen frequency compared with published results.

Research study	Rhesus antigen					No. of subjects	Country
	C (%)	c (%)	D (%)	E (%)	e (%)		
Present study	30.7	98.5	97.7	39.2	95.4	130	Nigeria
Jeremiah and Buseri, 2003 [16]	17.7	99.8	95.0	20.5	98.7	400	Nigeria
Jeremiah and Odumody, 2005 [17]	2.8	100.0	94.4	18.9	95.6	720	Nigeria
Gwaram and Abdullah, 2013 [15]	28.2	85.4	97.1	34.0	96.1	103	Nigeria
Hamed et al., 2013 [18]	42.7	94.0	93.6	14.0	98.2	2094	Mauritania
Bogui et al., 2014 [19]	22.0	99.9	92.9	13.8	99.9	651	Cote d'Ivoire

**Table 4:** Comparison of rhesus phenotype of the studied population with published data from other studies in Nigeria.

Rhesus phenotype		Present study (%)	Jeremiah and Buseri, 2003 [16] (%)	Jeremiah and Odumody, 2005 [17] (%)
Fisher notation	Weiner shorthand			
Rhesus positive				
cDe/cDe	R_0_R_0_	46.2	60.8	73.6
cDe/CDe	R_0_R_1_	12.3	14.5	1.9
cDE/cde	R_2_r	20.6	—	—
CDe/CDe	R_1_R_1_	0.8	—	—
cDE/Cde	R_2_r^1^	13.8	—	—
cDE/cDE	R_2_R_2_	2.3	—	4.4
cDE/CDE	R_2_R_z_	2.3	—	—
CDE/CDE	R_z_R_z_	0.8	—	—
cDE/cDe	R_2_R_0_	—	17.5	13.9
CDe/cDE	R_1_R_2_	—	1.8	—
cDE/CdE	R_2_r^y^	—	1.0	—
CDe/Cde	R_1_r^1^	—	0.2	—
CDe/CdE	R_1_r^y^	—	0.2	—
cDe/CDE	R_0_R_z_	—		0.6
Rhesus negative				
Cde/cde	r^1^r	0.8	1.0	0.3
cde/cde	Rr	0.8	3.0	5.3

## Data Availability

Datasets generated and analyzed in this study are available from the corresponding author upon request.

## References

[1] Mitra R., Mishra N., Rath G. P. (2014). Blood groups systems. *Indian Journal of Anaesthesia*.

[2] Lögdberg L., Reid M. E., Lamont R. E., Zelinski T. (2005). Human blood group genes 2004: chromosomal locations and cloning strategies. *Transfusion Medicine Reviews*.

[3] Okoroiwu H. U., Okafor I. M. (2018). Demographic characteristics of blood components transfusion recipients and pattern of blood utilization in a tertiary health institution in southern Nigeria. *BMC Hematology*.

[4] Kulkarni S. S., Snehalatha S., Desai P. K. (2002). Genetics of Rh blood group system. *Recent Trends in Transfusion Medicine*.

[5] Gundrajukuppam D. K., Vijaya S. K., Rajendran A., Sarella J. D. (2016). Prevalence of principal Rh blood group antigens in blood of the blood banle of tertiary care hospital in southern India. *Journal of Clinical and Diagnostic Research*.

[6] Westhoff C. M. (2004). The Rh blood group system in review: a new face for the next decade. *Transfusion*.

[7] Race R. R. (1948). The Rhesus phenotype and Fischer’s theory. *Blood*.

[8] Knowles S., Reagan F. (2006). Blood cell antigens: erythrocytes. *Lewis Practical Hematology*.

[9] World Health Organization (WHO) (1977). Twenty eighth report of WHO expert committee on biological standardization.

[10] Wiler M., Haimening D. M. (1998). The Rh blood group system. *Modern Blood Banking and Transfusion Practices*.

[11] Wagner F. F., Frohmajer A., Ladewig B. (2000). Weak D alleles express distinct phenotypes. *Blood*.

[12] David-West A. S. (1981). Blood transfusion and blood bank management in a tropical country. *Clinics in Haematology*.

[13] Hopkins D. F. (1969). Rhesus phenotypes and Rh(D) haemolytic disease of the newborn. *Vox Sanguinis*.

[14] Contreras M., Lubenko A. L., Hoffbrand A. V., Lewis S. M., Tuddenham (2001). Antigens in human blood. *Postgraduate Hematology*.

[15] Gwaram B. A., Abdullah S. (2013). Prevalence of Rh phenotypes among blood donors in Kano, Nigeria. *Journal of Medicine in the Tropics*.

[16] Jeremiah Z. A., Buseri F. I. (2003). Rh antigens and phenotype frequencies and probable genotypes for the four main ethnic groups in Port Harcourt, Nigeria. *Immunohematology*.

[17] Jeremiah Z. A., Odumody C. (2005). Rh antigens and phenotype frequencies of Ibibio, Efik and Ibo ethnic nationalities in Calabar, Nigeria. *Immunohematology*.

[18] Hamed C. T., Bollahi M. A., Abdelhamid I. (2013). Distribution of rhesus and kell blood group frequencies in Mauritanian population. *Blood Transfusion*.

[19] Bogui L. S., Dembele B., Sekongo Y., Abisse S., Konafe S., Sombo M. (2014). Phenotypic profile of Rh and kell blood group systems among blood donors in Cote d’Ivoire, west Africa. *Journal of Blood Transfusion*.

[20] Ebimgbo S. O., Okoye U. O. Promoting cultural diversity for sustainable development in Nigeria. The role of social workers.

[21] Dean L. (2005). *Blood Group and Red Cell Antigens*.

[22] Daniels G. L. (2002). *Human Blood Groups*.

[23] Hackney D. N., Knudtson E. J., Rossi K. Q., Krugh D., O’Shaughnessy R. W. (2004). Management of pregnancies complicated by anti-c isoimmunization. *Obstetrics & Gynecology*.

[24] Appelman Z., Lurie S., Juster A., Borenstein R. (1990). Severe hemolytic disease of the newborn due to anti-c. *International Journal of Gynecology & Obstetrics*.

[25] Reid M. E., Lomas-Francis C. (2004). *The Blood Group Antigens Facebook*.

[26] Makroo R., Gupta R. R., Bhatia A., Rosamma N. L. (2014). Rhesus Phenotype, allele and haplotype frequencies among 51,857 blood donors in north India. *Blood Transfusion*.

[27] Wagner F. F., Kasulke D., Kerowgan M., Flegel W. A. (1995). Frequencies of the blood groups ABO, rhesus, D category VI, kell, and of clinically relevant high-frequency antigens in south-western Germany. *Transfusion Medicine and Hemotherapy*.

[28] Joshi S., Vasantha K. (2012). A profile of rare bloods in India and its impact in blood transfusion service. *Asian Journal of Transfusion Science*.

[29] Reesink H. W., Engelfriet C. P., Schennach H. (2008). Donors with a rare pheno (geno) type. *Vox Sanguinis*.

[30] Enosolease M., Bazuaye G. (2008). Distribution of ABO and Rh-D blood groups in the Benin area of Niger-Delta: implication for regional blood transfusion. *Asian Journal of Transfusion Science*.

